# Ambient Air Pollution and Out-of-Hospital Cardiac Arrest in Beijing, China

**DOI:** 10.3390/ijerph14040423

**Published:** 2017-04-14

**Authors:** Ruixue Xia, Guopeng Zhou, Tong Zhu, Xueying Li, Guangfa Wang

**Affiliations:** 1 Department of Respiratory Medicine, First Hospital, Health Sciences Centre, Peking University, 8 Xishiku Rd., Beijing 100034, China; xiayiyuan@bjmu.edu.cn; 2 Department of Hospital Information, First Hospital, Health Sciences Centre, Peking University, 8 Xishiku Rd., Beijing 100034, China; zhougp@163.com (G.Z.); xyinglee@163.com (X.L.); 3 State Key Laboratory of Environmental Simulation and Pollution Control, College of Environmental Sciences and Engineering and Centre for Environment and Health, Peking University, Beijing 100871, China; tzhu@pku.edu.cn

**Keywords:** air pollution, out-of-hospital cardiac arrest, case-crossover study, Beijing, fine particulate matter

## Abstract

Air pollutants are associated with cardiovascular death; however, there is limited evidence of the effects of different pollutants on out-of-hospital cardiac arrests (OHCAs) in Beijing, China. We aimed to investigate the associations of OHCAs with the air pollutants PM_2.5–10_ (coarse particulate matter), PM_2.5_ (particles ≤2.5 μm in aerodynamic diameter), nitrogen dioxide (NO_2_), sulfur dioxide (SO_2_), carbon monoxide (CO), and ozone (O_3_) between 2013 and 2015 using a time-stratified case-crossover study design. We obtained health data from the nationwide emergency medical service database; 4720 OHCA cases of cardiac origin were identified. After adjusting for relative humidity and temperature, the highest odds ratios of OHCA for a 10 μg/m^3^ increase in PM_2.5_ were observed at Lag Day 1 (1.07; 95% confidence interval (CI): 1.04–1.10), with strong associations with advanced age (aged ≥70 years) (1.09; 95% CI: 1.05–1.13) and stroke history (1.11; 95% CI: 1.06–1.16). PM_2.5–10_ and NO_2_ also showed significant associations with OHCAs, whereas SO_2_, CO, and O_3_ had no effects. After simultaneously adjusting for NO_2_ and SO_2_ in a multi-pollutant model, PM_2.5_ remained significant. The effects of PM_2.5_ in the single-pollutant models for cases with hypertension, respiratory disorders, diabetes mellitus, and heart disease were higher than those for cases without these complications; however, the differences were not statistically significant. The results support that elevated PM_2.5_ exposure contributes to triggering OHCA, especially in those who are advanced in age and have a history of stroke.

## 1. Introduction

Cardiac arrest is defined as the cessation of cardiac mechanical activity and the subsequent cessation of blood circulation [1] and mostly occurs outside hospitals [2]. Despite extensive spending in an effort to introduce novel devices and drugs, the low survival rate of out-of-hospital cardiac arrest (OHCA) (<7.6%) has not significantly improved over the last 30 years [3]. Therefore, OHCA has become a considerable public health burden worldwide [4], particularly in China, where the success rate of heartbeat restoration is less than 0.4% [5]. Consequently, discussion of the prediction of OHCA incidence and screening subjects at risk is inevitable.

Recent epidemiological evidence has shown that increased sudden cardiovascular mortality is associated with high air pollution levels [6]; therefore, OHCAs may be an important part of the association between air pollution and cardiovascular disease-related death. Due to rapid global economic growth and motorization, air pollution has become a common environmental problem worldwide. In China, air pollution levels have been increasing rapidly and have reached the highest levels in the world [7]. Thus, understanding the impacts of air pollutants on OHCAs is important. The effects of air pollutants on cardiovascular mortality have been assessed in developed countries, e.g., the U.S. [8,9], European countries [10,11,12,13], and Australia [14,15], but the findings are inconsistent and vary greatly by location. Therefore, more research is needed in countries with high concentration levels of air pollutants.

Some traditional risk factors for cardiovascular disease might trigger OHCA onset, including heavy physical activity, psychosocial triggers, seasonal variation, overeating, and respiratory infection [16]. However, ambient air pollution requires attention because it is ubiquitous and involuntary. Furthermore, the influence of air pollutants on cardiovascular mortality can be modified by individual characteristics, including sex, age, and history of disease [17]. Air pollutant levels and individual characteristics may have interactive effects on the estimates of OHCAs. However, in China, studies of the effects of air pollution on OHCA are lacking despite the fact that they are needed to develop OHCA warning systems to reduce associated adverse health impacts.

In this study, we used the EMS (emergency medical service) database to examine the influence of exposure to different ambient air pollutants on OHCAs among residents of Beijing, China, and identify individuals who are more susceptible to elevated PM_2.5_ (particles ≤2.5 μm in aerodynamic diameter) exposure.

## 2. Materials and Methods

### 2.1. Study Population

This study was conducted in Beijing, the capital city of China (located in Northern China at a latitude of 39°26′–41°03′ N and covering an area of 1369 km^2^), which has approximately 13.5 million permanent residents. Using the EMS database, we obtained information on patients who experienced an OHCA between January 2013 and December 2015 (1094 days) and were treated by EMS personnel. The database has been described in detail elsewhere [18]; only individuals who had Beijing ID cards were included. We reviewed EMS reports, death certificates, and autopsy reports (where available) to exclude cases of death due to trauma, drowning, poisoning, asphyxia, electric shock, or unknown causes (Figure S1). Then, we collected the patients’ medical histories from the medical insurance system in Beijing, including history of hypertension, respiratory disorder, diabetes mellitus, heart disease, and stroke as well as other relevant information, such as age and sex. We excluded cases with values missing from the database. We recorded the subject-related information in a manner that protected individual identities. This study was approved by the Review Board of Peking University Research Subjects Ethics (No. 2016 (1027)).

### 2.2. Air Pollutants and Meteorological Data

The daily 24 h air pollution concentrations were obtained from all 17 monitoring stations [19] in Beijing operated by the China National Environmental Monitoring Center and from a U.S. Embassy monitoring station in Beijing (Figure S2). All stations monitored PM_2.5_, coarse particulate matter (PM_2.5–10_), and gaseous pollutants, including sulfur dioxide (SO_2_), nitrogen dioxide (NO_2_), carbon monoxide (CO), and ozone (O_3_), on an hourly basis. We calculated the daily mean concentrations of the pollutants. All stations had data for daily periods of 20–24 h, and no data were missing. We obtained the meteorological data, including the daily mean relative humidity (RH) and temperature, from the Meteorological Data Sharing Service System in China (World Meteorological Organization) station 54511, located at 39°48′ N, 116°28′ E, in the southeastern part of Beijing.

### 2.3. Statistical Analysis

We used a time-stratified case-crossover design to analyze OHCA events, air pollutants and meteorological data. This design was introduced by Maclure [20] to estimate rare acute episodic events following short-term exposure with transient effects. In addition, this design can be regarded as a special type of case-control study. Specifically, the control periods are the days in which a patient does not experience an OHCA on the same day of the week (DOW) in the same month and year as the case. Thus, there were theoretically 3–4 control days per case day [10,21]. This approach controls for confounders such as the DOW, monthly trends, seasonal trends, and long-term trends by design and can also minimize the possibility of short-term time invariant potential confounders (such as age, health history, and socio-economic status) in the exposure variables. A conditional logistic regression analysis was performed to estimate the association of ambient pollutants with OHCA events while controlling for exposure to meteorological factors, i.e., temperature (natural cubic spline, 3 knots) and RH (natural cubic spline, 3 knots) on concurrent OHCA days. The numbers of knots and lags of meteorological factors were chosen by comparing Akaike’s Information Criterion (AIC) values; the result showed that the models always have smaller AIC values and larger estimated. We also calculated the odds ratios (ORs) and the 95% confidence intervals (CIs) per 10 μg/m^3^ increase in pollutants levels. Furthermore, we statistically compared the concentrations on control days with the daily average concentrations on the onset day (Lag 0) and on Days 1–5 before onset (Lags 1, 2, 3, 4, and 5). In addition, we ran multi-pollutant models with PM_2.5_ + PM_2.5–10_, PM_2.5_ + SO_2_, PM_2.5_ + NO_2_, and PM_2.5_ + SO_2_ + NO_2_ and with PM_2.5–10_ + SO_2_, PM_2.5–10_ + NO_2_, and PM_2.5–10_ + SO_2_ + NO_2_ to estimate the effect of one pollutant (PM_2.5_ or PM_2.5–10_) while controlling for the effects of the others. Because the concentrations of CO were extremely low in Beijing, we did not include this pollutant in the multi-pollutant modelling analyses, which were constructed to estimate the independent effects of PM_2.5_ and PM_2.5–10_ among the other pollutants. Further, we calculated the interaction terms for PM_2.5_ concentrations and some potential effect modifiers (e.g., age × PM_2.5_; history of stroke × PM_2.5_) for the same conditional logistic regression model described above (only for Lag 1). We defined the season (cold season: October–March; warm season: April–September) in accordance with a previous study in Beijing [22]. 

We then conducted sensitivity analyses to determine the robustness of the results as follows: (1) We used a different method to select a control day for cases: we matched the temperature to within 0.5 °C on both days and chose the control day that was at least 3 days apart from the case day to avoid short-term autocorrelations [23]. (2) We used moving average concentrations of pollutants instead of single daily average concentrations in the single- and multi-pollutant models of Lag Days 0–5 (Lags 0–5) to examine the association of air pollutants and OHCAs. We also controlled for mean temperature and RH on the current day, according to smaller AIC values and larger estimates of the result.

The logistic regression analysis was conducted in R version 2.12.2 (25 February 2011; R Development Core Team, Vienna, Austria). A two-sided level of significance of 0.05 was used to indicate statistical significance.

## 3. Results

### 3.1. Characteristics of the Study Population

The characteristics of the study subjects are shown in Table 1. A total of 5851 OHCA cases from January 2013 to December 2015 (1094 days) were recorded in Beijing (Figure S1). The following exclusion criteria were used: death due to trauma (*n* = 206), poisoning (*n* = 232), drowning (*n* = 121), electric shock (*n* = 124), asphyxia (*n* = 209), or unknown causes (*n* = 239). After excluding subjects, a total of 4720 OHCAs were analyzed in this study. The mean (±standard deviation, SD) age of the study population was 64.9 (±15.0) years. Approximately 57.0% of subjects were older than 70 years of age, and 60.6% were males. The proportions of OHCA cases with a medical history of hypertension, respiratory disorder, diabetes mellitus, heart disease, and stroke were 32.3%, 24.6%, 27.0%, 35.7%, and 17.3%, respectively, and approximately 56.7% experienced OHCA during the cold season (Table 1).

### 3.2. Air Pollution and Incidence of OHCAs in Single- and Multi-Pollutant Models

**Figure 1:**
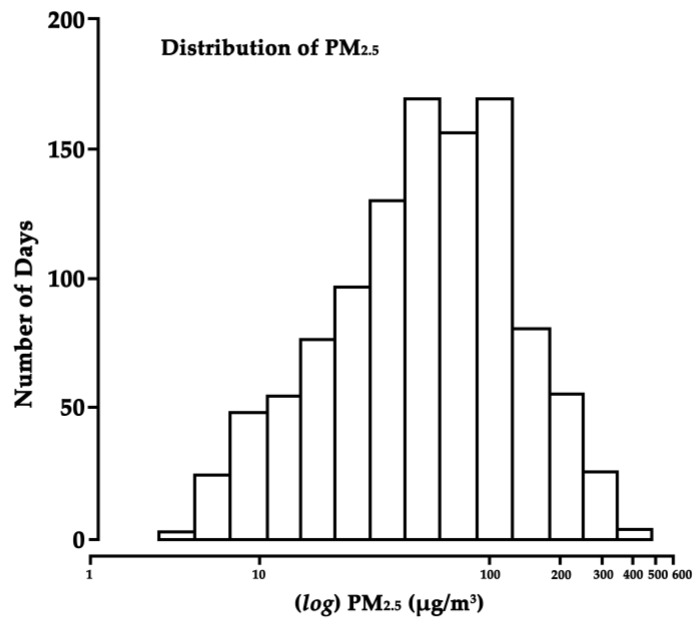
Distribution of PM_2.5_ levels in Beijing, China, 2013–2015. PM_2.5_ on the *X*-axis is presented on a logarithmic scale.

The histogram in Figure 1 shows that there was a wide range (5.0 to 476.0 µg/m^3^) of ambient PM_2.5_ concentrations during the study. Table 2 shows a statistical summary of air pollution and meteorological variable levels during the study period. The IQRs (interquartile ranges) of air pollutants were 75.0 μg/m^3^ for PM_2.5_, 88.5 μg/m^3^ for PM_2.5–10_, 29.8 μg/m^3^ for NO_2_, 0.9 mg/m^3^ for CO, 15.2 μg/m^3^ for SO_2_, and 55.8 μg/m^3^ for O_3_. On 122 days, approximately 11% of the observed daily average PM_2.5_ concentrations were ≤15 μg/m^3^, which is classified as “good” by the U.S. Environmental Protection Agency [24]. On 123 days, the daily average PM_2.5_ concentrations were >150 μg/m^3^, which is considered “very unhealthy.” The Spearman correlation coefficients for daily air pollutant concentrations, temperatures, and RH are shown in Table S1. PM_2.5_ was highly correlated with PM_2.5–10_ (*r* = 0.89), moderately correlated with SO_2_ (*r* = 0.63), NO_2_ (*r* = 0.58) and CO (*r* = 0.49), and negatively correlated with O_3_ (*r* = −0.13) over the study period.

**Figure 2:**
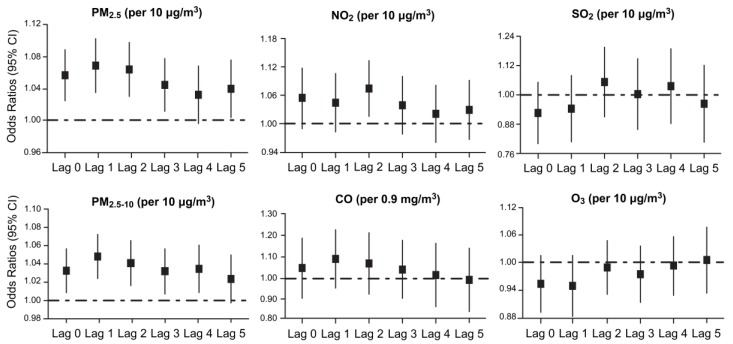
The ORs of OHCA per 10 µg/m^3^ increase in pollutant concentrations (per interquartile range increase, 0.9 mg/m^3^ in CO) in single-pollutant models at Lag 0 to Lag 5.

Figure 2 shows the ORs of OHCA in single-pollutant models using various lag lengths. When we examined the effects of each pollutant at Lag 0 to Lag 5, PM_2.5_, PM_2.5–10_, and NO_2_ exposure were associated with an increased risk of OHCA, whereas SO_2_, CO, and O_3_ exposure were not associated with an increased risk of OHCA. The strongest effects observed in the distributed lag model in response to a 10 µg/m^3^ increase in exposure were 1.07 (95% CI: 1.04 to 1.10) for PM_2.5_ at Lag 1, 1.05 (95% CI: 1.03 to 1.07) for PM_2.5–10_ at Lag 1, and 1.08 (95% CI: 1.02 to 1.13) for NO_2_ at Lag 2. We observed that the PM_2.5_ and PM_2.5–10_ increased the risk of OHCA at Lags 0–3, with a general downward trend in ORs with Lags 1–5. Periods longer than Lag 5 did not show strong relationships with OHCA for each pollutant. 

Table 3 shows the OR estimates derived from single- and multi-pollutant models measured at Lag 1. In the two-pollutant model, the estimated association between PM_2.5_ and OHCA was attenuated after adjustment for PM_2.5–10_ in comparison with the single-pollutant model, but generally, the association between PM_2.5_ and OHCA remained significant. In the multi-pollutant analysis, the estimates for PM_2.5_ and PM_2.5–10_ were slightly increased after adjustment for SO_2_ but were reduced after adjustment for NO_2_ and SO_2_ simultaneously. 

### 3.3. Subgroup Analysis

**Figure 3:**
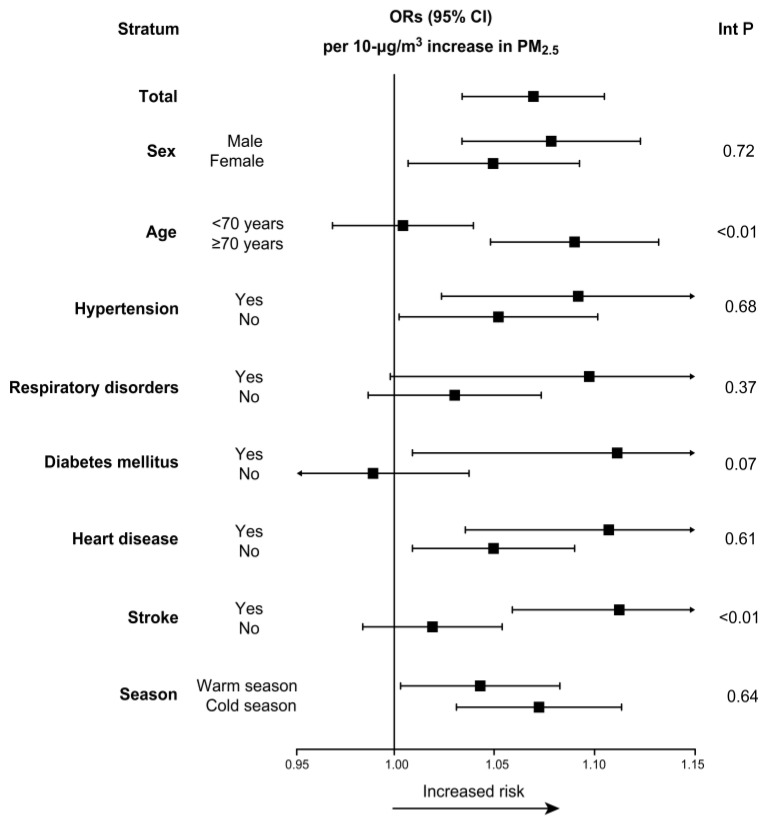
Results of subgroup analyses for PM_2.5_ (per 10 μg/m^3^) at a lag of 1 day (The *X*-axis represents ORs with 95% CIs. Abbreviations: Int P: interaction *p*-value).

Figure 3 shows the results of analyses for PM_2.5_ concentrations per 10 µg/m^3^ increase at Lag 1, stratified by potential effect modifiers. Analysis of the cases stratified by demographic characteristics showed that the risk from exposure to PM_2.5_ was highest for those ≥70 years of age (1.09; 95% CI: 1.05 to 1.13) and that the effect estimate for PM_2.5_ was higher for male subjects. Analysis of the cases stratified by a history of disease showed that those with stroke history were more susceptible to PM_2.5_ (1.11; 95% CI: 1.06 to 1.16), and this was the strongest effect observed. The interaction was significant for the age and stroke history subgroups (*p* < 0.01). The incidence of OHCA after exposure to PM_2.5_ was higher in individuals with hypertension, respiratory disorders, diabetes mellitus, or a history of heart disease than in those without these complications, but these differences were statistically insignificant. The effect estimate for PM_2.5_ was higher in the cold season than in the warm season, but this difference was not significant.

### 3.4. Sensitivity Analysis

For the sensitivity analysis, the results did not change substantially after choosing the control days by matching the temperatures to within 0.5 °C in the case-crossover models (Figure S3). The strongest association with OHCAs was observed for Day Lag 2 of NO_2_ (OR = 1.08; *p* < 0.01) for the model matching the DOW but for Lag Day 3 (OR = 1.09; *p* < 0.01) for the model matching the mean temperature within 0.5 °C. Furthermore, the ORs associated with an increased risk of OHCAs using the moving-average concentrations (Lags 0–5) of PM_2.5_ and PM_2.5–10_ were similar to the results for the daily average concentrations (Lags 0–5) (Tables S2 and S3). 

## 4. Discussion

By analyzing the incidence of OHCA in this study, we found that air pollutants levels were significantly associated with OHCA events (Figure 2). Among all examined pollutants, PM_2.5_ and PM_2.5–10_ were most strongly associated with OHCAs; NO_2_, albeit not as strongly as PM_2.5_ and PM_2.5–10_, was also associated with OHCA. There was no positive correlation between OHCA events and SO_2_, CO, or O_3_ for any lag period. By contrast, in the U.S. [9] and Finland [13], the acute effects per IQR increase in the pollutant levels of PM on OHCAs were found to be small (0.3–1.1% and 1–7%, respectively, excess relative risk) compared with the effects of O_3_ (3.4–4.6% and 9–18%, respectively). These differences may be due to the differences in major air pollutants between developed countries and Beijing. Ozone is often the result of an atmosphere photochemical reaction involving natural oxygen molecules and solar radiation. Ozone is the main air pollutant and is often excessive in cities of developed countries with high visibility and low PM levels. However, in Beijing, PM is the main air pollutant due to anthropogenic activities involving the combustion of coal and petroleum products [25].

In addition, despite the theoretical statistical risks ascribed to all residents in this study, the increased risks from PM_2.5_ exposure are not equally distributed within the population. The interaction analysis revealed that a history of stroke influenced the association between PM_2.5_ and OHCA; this has not been previously reported and was unexpected based on previous reports of other Asian cities [26]. Patients with a history of stroke were considered the susceptible cases in this study, possibly because such patients are more likely to represent the individuals with “vulnerable plaque” or “vulnerable circulation.” The acute physiological responses that occur after PM_2.5_ inhalation are partly mediated by autonomic nervous system-mediated changes, such as vasoconstriction, increases in blood pressure increasing and arrhythmias, along with the direct effects of air pollutant constituents on platelets (e.g., procoagulant and thrombotic changes) [17]. In addition, the biological inflammatory consequences induced by PM_2.5_ inhalation, such as the elevation of white blood cell counts and cytokine levels, increase vulnerability to atherosclerotic plaques, enhanced coagulation, thrombus formation, and even arrhythmia in chronically vulnerable individuals [17]. Consequently, it is reasonable to assume that patients with a history of stroke may be more sensitive to air pollution and more prone to cardiac death.

We also examined the difference in PM_2.5_-related OHCAs between cases with hypertension or heart disease and those without. The effect of PM_2.5_ was stronger in individuals with a history of hypertension or heart disease than in those without (Figure 3). These results are somewhat consistent with a recent study that analyzed data from the single-tiered EMS system in Korea [26], which showed that patients with impaired endothelial function might be more susceptible to ambient air pollution [27,28]. However, although the risks of OHCAs were higher in patients with hypertension or heart disease, the differences between the groups with and without a history of hypertension or heart disease were not significant, with *p*-values of 0.68 and 0.61, respectively. In our future studies, we aim to explore the responses to ambient air pollutants while considering additional individual characteristics and potentially important risk modifiers, such as smoking behavior, use of cardiac medications, and exercise. Triggering activities such as these may be correlated with cardiovascular mortality; however, the degree of confounding is expected to be limited. We will perform stratified analyses to determine if there is effect modification by these factors and further clarify the role of a history of hypertension or heart disease in OHCA risk.

In many previous epidemiological studies, the time windows for mortality [29] or other risk effects [30] of air pollution were days to years. However, the findings of this study highlight the significance of a shorter time window for OHCAs. This rapid response is evident from the occurrence of the highest OR at Lag Day 1 versus Lag Day 2 to Lag Day 5 (Figure 2), consistent with previous reports of a rapid-acting effect of air pollutants on the cardiovascular system [17]. For example, Rich et al. [31] studied 125 healthy young adults during the Beijing Olympics and revealed that short-term exposure to air pollution led to acute inflammatory and pro-thrombotic responses in the early lag period. Furthermore, Pope et al. [32] found that PM_2.5_ exposure was associated with increased endothelial cell apoptosis within 1–48 h. In addition, some studies have shown that short-term exposure to PM_2.5_ might cause acute cardiovascular stress, thereby increasing blood pressure, triggering atrial fibrillation, and eventually contributing to atherogenesis, cardiac arrest and even acute cardiovascular mortality [33]. These findings may partly reveal the underlying biological mechanisms, including potentially different mechanisms between cardiac arrest and other chronic cardiovascular endpoints, such as coronary artery calcification and changes in intima thickness.

The magnitude of the effect of PM_2.5_ on OHCA varied among studies [34]. For example, in a case-crossover study [10] of 4657 OHCA patients and outdoor levels of PM_2.5_ in Copenhagen, the strongest association with OHCAs was observed for Lag Day 4 of PM_2.5_; the percentage increase in risk for an IQR increase of 4.69 µg/m^3^ was 5.2% (95% CI: 1.0, 9.5%). Rosenthal et al. [8] found that the risk of OHCA in association with PM_2.5_ was much higher in individuals who were white, 60–75 years of age, or suffering from asystole (OR = 1.18, 1.25 or 1.22 per 10 µg/m^3^ increase in PM_2.5_; *p* < 0.05) in Indianapolis, Indiana. A study conducted in Melbourne [14] investigated the daily PM_2.5_ concentrations and risk of OHCA in 8434 cases from 2003 through 2006 and found that an IQR increase of 4.26 µg/m^3^ over 2 days was associated with an increase in OHCA risk of 3.6% (95% CI: 1.3, 6.0%); the most susceptible individuals to PM_2.5_ exposure in the study were 65–74 years of age. Another case-crossover study with a large sample size in Houston, Texas [9] of 11,677 OHCA events between 2004 and 2011 reported that an average increase of 6 µg/m^3^ in PM_2.5_ at Lag Day 2 was associated with an increased risk of OHCA (1.046; 95% CI: 1.012, 1.082). We presume that these inter-study differences may be due to the following: (1) study differences in the population characteristics and proportions of cases with various pre-existing cardiovascular diseases (e.g., the prevalence of cardiovascular disease is lower in China than in Western countries [35]); (2) differences in particulate matter composition of air pollution between Beijing and areas in developed countries [36]; and (3) differences in age structure. In the present study, we selected all age groups of study subjects, whereas previous studies have targeted elderly persons.

This study has several strengths. The most important is that this is the first study conducted in the capital city of Beijing, China, to investigate the association between OHCA risk and exposure to air pollutants. In addition, there was a well-defined outcome regarding both etiology and timing in the large sample, with more than 4500 events spanning a 3-year period. Furthermore, the study used patient medical records retrospectively and identified risk factors that increased patient susceptibility to harm from air pollution.

There are also some limitations in our study. We analyzed the air pollution data from fixed monitoring sites as an exposure estimate for the entire population, assuming that the exposure is homogeneous across the whole area. While possibly introducing non-differential misclassification of personal exposure, this analysis would underestimate any association. However, in ecological studies, using levels of daily concentrations of air pollutants (averaged over all available stations) as measures of exposure is considered acceptable [37]. Furthermore, the patient data were from Beijing local residents, and the individuals from the floating population were not involved, potentially leading to underestimation of the true relative risk of OHCA because the proportion of elderly residents in our study was higher than that in the floating population [38]. In addition, only complete OHCA data were used in this study; we had no data regarding other risk factors, such as body weight, smoking history, etiology of cardiac arrest, and history of cardiac medication use, as available data were limited. Therefore, the direction and magnitude of these potential biases are unknown. Finally, although the models were designed for OHCA acute events, there may also be long-term chronic exposure aspects of this influence; we could use models such as Cox proportional hazards models [39] to estimate the associations between time-dependent PM_2.5_ exposure and OHCA-related mortality in future studies.

## 5. Conclusions 

The results of this study confirm the link between OHCA and PM_2.5_ in Beijing, China, and introduce evidence of a similar link with PM_2.5–10_. Further, people of advanced age or with a history of stroke were more susceptible to the adverse effects of PM_2.5_ for OHCAs. Exposure to SO_2_, CO, and O_3_ was not associated with OHCA in our study.

## Figures and Tables

**Table 1:** Characteristics of cases.

Risk Factor	*N* = 4720
Age	
<35 years (%)	1.7
35–70 years (%)	41.3
≥70 years (%)	57.0
Sex	
Male (%)	60.6
Female (%)	39.4
Medical history	
Hypertension (%)	32.3
Respiratory disorders (%)	24.6
Diabetes mellitus (%)	27.0
Heart disease (%)	35.7
Stroke (%)	17.3
Season of onset	
Warm (April to September, %)	43.3
Cold (October to March, %)	56.7

**Table 2:** Distribution of the daily mean meteorological data and air pollutant concentrations in Beijing, China, 2013–2015.

Daily Data	Mean	SD	Min	P (25)	Median	P (75)	Max
Meteorological							
Temperature, °C	13.2	11.1	−10.0	4.0	12.5	23.5	31.0
Relative humidity, %	68.2	16.8	39	55	68	71	86
Air pollutants							
PM_2.5_, μg/m^3^	76.0	65.8	5.0	29.0	58.0	104.0	476.0
PM_2.5–10_, μg/m^3^	96.4	72.9	18.2	45.0	87.2	133.5	481.2
O_3_, μg/m^3^	61.8	22.1	12.1	24.2	50.1	80.0	169.5
NO_2_, μg/m^3^	51.7	7.8	6.2	31.3	43.4	61.1	136.2
CO, mg/m^3^	1.20	0.28	0.22	0.61	0.94	1.47	8.11
SO_2_, μg/m^3^	14.4	4.3	2.2	3.1	7.1	18.3	133.1

**Table 3:** OR estimates of OHCA per 10 μg/m^3^ increase in air pollutants at a lag of 1 day.

Pollutant Measure	OR	95% CIs
Single-pollutant models		
PM_2.5_, unadjusted	1.07	1.04–1.10
PM_2.5–10_, unadjusted	1.05	1.03–1.07
NO_2_, unadjusted	1.05	0.98–1.11
SO_2_, unadjusted	0.95	0.82–1.08
Multi-pollutant models		
PM_2.5_, adjusted for PM_2.5–10_	1.04	1.01–1.06
PM_2.5_, adjusted for NO_2_	1.07	1.03–1.11
PM_2.5_, adjusted for SO_2_	1.08	1.04–1.12
PM_2.5_, adjusted for NO_2_, SO_2_	1.04	0.99–1.09
PM_2.5–10_, adjusted for NO_2_	1.03	1.00–1.07
PM_2.5–10_, adjusted for SO_2_	1.06	1.01–1.11
PM_2.5–10_, adjusted for NO_2_, SO_2_	1.02	0.99–1.05

## Supplementary Materials

The following are available online at www.mdpi.com/1660-4601/14/4/423/s1, Figure S1: Flow diagram of the study population. Figure S2: The distribution of all 17 air pollutant monitoring stations and the U.S. Embassy monitoring station located in urban areas in Beijing, China. Figure S3: Sensitivity analyses performed using case-crossover models, with estimates from control days matched by temperature to within 0.5 °C. Table S1: Spearman correlation coefficients among air pollutants, temperatures and relative humidity in Beijing during 2013–2015. Table S2: Estimates of OHCA for 10 µg/m^3^ moving average of PM_2.5_ concentrations increase in single- and multi-pollutant models at Lag 0 to Lag 5. Table S3: Estimates of OHCA for 10 µg/m^3^ moving average of PM_2.5–10_ concentrations increase in single- and multi-pollutant models at Lag 0 to Lag 5.

## References

[1] Mozaffarian D., Benjamin E.J., Go A.S., Arnett D.K., Blaha M.J., Cushman M., Das S.R., de Ferranti S., Despres J.P., Writing Group Members (2016). Heart disease and stroke statistics-2016 update: A report from the American Heart Association. Circulation.

[2] Zheng Z.J., Croft J.B., Giles W.H., Mensah G.A. (2001). Sudden cardiac death in the United States, 1989 to 1998. Circulation.

[3] Sasson C., Rogers M.A., Dahl J., Kellermann A.L. (2010). Predictors of survival from out-of-hospital cardiac arrest: A systematic review and meta-analysis. Circ. Cardiovasc. Qual. Outcomes.

[4] Berdowski J., Berg R.A., Tijssen J.G., Koster R.W. (2010). Global incidences of out-of-hospital cardiac arrest and survival rates: Systematic review of 67 prospective studies. Resuscitation.

[5] Gu X.M., Li Z.H., He Z.J., Zhao Z.W., Liu S.Q. (2016). A meta-analysis of the success rates of heartbeat restoration within the platinum 10 min among outpatients suffering from sudden cardiac arrest in China. Mil. Med. Res..

[6] GBD 2015 Risk Factors Collaborators (2016). Global, regional, and national comparative risk assessment of 79 behavioural, environmental and occupational, and metabolic risks or clusters of risks, 1990–2015: A systematic analysis for the global burden of disease study 2015. Lancet.

[7] Van Donkelaar A., Martin R.V., Brauer M., Kahn R., Levy R., Verduzco C., Villeneuve P.J. (2010). Global estimates of ambient fine particulate matter concentrations from satellite-based aerosol optical depth: Development and application. Environ. Health Perspect..

[8] Rosenthal F.S., Carney J.P., Olinger M.L. (2008). Out-of-hospital cardiac arrest and airborne fine particulate matter: A case-crossover analysis of emergency medical services data in Indianapolis, Indiana. Environ. Health Perspect..

[9] Ensor K.B., Raun L.H., Persse D. (2013). A case-crossover analysis of out-of-hospital cardiac arrest and air pollution. Circulation.

[10] Wichmann J., Folke F., Torp-Pedersen C., Lippert F., Ketzel M., Ellermann T., Loft S. (2013). Out-of-hospital cardiac arrests and outdoor air pollution exposure in Copenhagen, Denmark. PLoS ONE.

[11] Milojevic A., Wilkinson P., Armstrong B., Bhaskaran K., Smeeth L., Hajat S. (2014). Short-term effects of air pollution on a range of cardiovascular events in england and wales: Case-crossover analysis of the minap database, hospital admissions and mortality. Heart.

[12] Raza A., Bellander T., Bero-Bedada G., Dahlquist M., Hollenberg J., Jonsson M., Lind T., Rosenqvist M., Svensson L., Ljungman P.L. (2014). Short-term effects of air pollution on out-of-hospital cardiac arrest in Stockholm. Eur. Heart J..

[13] Rosenthal F.S., Kuisma M., Lanki T., Hussein T., Boyd J., Halonen J.I., Pekkanen J. (2013). Association of ozone and particulate air pollution with out-of-hospital cardiac arrest in Helsinki, Finland: Evidence for two different etiologies. J. Expo. Sci. Environ. Epidemiol..

[14] Dennekamp M., Akram M., Abramson M.J., Tonkin A., Sim M.R., Fridman M., Erbas B. (2010). Outdoor air pollution as a trigger for out-of-hospital cardiac arrests. Epidemiology.

[15] Straney L., Finn J., Dennekamp M., Bremner A., Tonkin A., Jacobs I. (2014). Evaluating the impact of air pollution on the incidence of out-of-hospital cardiac arrest in the perth metropolitan region: 2000–2010. J. Epidemiol. Community Health.

[16] Tofler G.H., Muller J.E. (2006). Triggering of acute cardiovascular disease and potential preventive strategies. Circulation.

[17] Brook R.D., Rajagopalan S., Pope C.A., Brook J.R., Bhatnagar A., Diez-Roux A.V., Holguin F., Hong Y., Luepker R.V., Mittleman M.A. (2010). Particulate matter air pollution and cardiovascular disease: An update to the scientific statement from the American Heart Association. Circulation.

[18] Shao F., Li C.S., Liang L.R., Li D., Ma S.K. (2014). Outcome of out-of-hospital cardiac arrests in Beijing, China. Resuscitation.

[19] Xu Q., Li X., Wang S., Wang C., Huang F., Gao Q., Wu L., Tao L., Guo J., Wang W. (2016). Fine particulate air pollution and hospital emergency room visits for respiratory disease in urban areas in Beijing, China, in 2013. PLoS ONE.

[20] Maclure M. (1991). The case-crossover design: A method for studying transient effects on the risk of acute events. Am. J. Epidemiol..

[21] Levy D., Lumley T., Sheppard L., Kaufman J., Checkoway H. (2001). Referent selection in case-crossover analyses of acute health effects of air pollution. Epidemiology.

[22] Zhang Q., Qi W., Yao W., Wang M., Chen Y., Zhou Y. (2016). Ambient particulate matter (PM_2.5_/PM_10_) exposure and emergency department visits for acute myocardial infarction in chaoyang district, Beijing, China during 2014: A case-crossover study. J. Epidemiol./Jpn. Epidemiol. Assoc..

[23] Scheers H., Mwalili S.M., Faes C., Fierens F., Nemery B., Nawrot T.S. (2011). Does air pollution trigger infant mortality in Western Europe? A case-crossover study. Environ. Health Perspect..

[24] U.S. Environmental Protection Agency (2006). Guidelines for the Reporting of Daily Air Quality—The Air Quality Index (AQI).

[25] Song Y., Wang X., Maher B.A., Li F., Xu C., Liu X., Sun X., Zhang Z. (2016). The spatial-temporal characteristics and health impacts of ambient fine particulate matter in China. J. Clean. Prod..

[26] Kang S.H., Heo J., Oh I.Y., Kim J., Lim W.H., Cho Y., Choi E.K., Yi S.M., Do Shin S., Kim H. (2016). Ambient air pollution and out-of-hospital cardiac arrest. Int. J. Cardiol..

[27] Chung J.W., Bang O.Y., Ahn K., Park S.S., Park T.H., Kim J.G., Ko Y., Lee S., Lee K.B., Lee J. (2017). Air pollution is associated with ischemic stroke via cardiogenic embolism. Stroke J. Cereb. Circ..

[28] Newman J.D., Thurston G.D., Cromar K., Guo Y., Rockman C.B., Fisher E.A., Berger J.S. (2015). Particulate air pollution and carotid artery stenosis. J. Am. Coll. Cardiol..

[29] Yang J., Zhou M., Yin P., Li M., Ou C.Q., Gu S., Liu Q. (2016). Mortality as a function of dust-haze in China: A multi-city time-series study. Lancet.

[30] Kaufman J.D., Adar S.D., Barr R.G., Budoff M., Burke G.L., Curl C.L., Daviglus M.L., Diez Roux A.V., Gassett A.J., Jacobs D.R. (2016). Association between air pollution and coronary artery calcification within six metropolitan areas in the USA (the multi-ethnic study of atherosclerosis and air pollution): A longitudinal cohort study. Lancet.

[31] Rich D.Q., Kipen H.M., Huang W., Wang G., Wang Y., Zhu P., Ohman-Strickland P., Hu M., Philipp C., Diehl S.R. (2012). Association between changes in air pollution levels during the Beijing Olympics and biomarkers of inflammation and thrombosis in healthy young adults. JAMA.

[32] Pope C.A., Bhatnagar A., McCracken J.P., Abplanalp W., Conklin D.J., O’Toole T. (2016). Exposure to fine particulate air pollution is associated with endothelial injury and systemic inflammation. Circ. Res..

[33] Link M.S., Luttmann-Gibson H., Schwartz J., Mittleman M.A., Wessler B., Gold D.R., Dockery D.W., Laden F. (2013). Acute exposure to air pollution triggers atrial fibrillation. J. Am. Coll. Cardiol..

[34] Zhao R., Chen S., Wang W., Huang J., Wang K., Liu L., Wei S. (2017). The impact of short-term exposure to air pollutants on the onset of out-of-hospital cardiac arrest: A systematic review and meta-analysis. Int. Cardiol..

[35] World Health Organization (WHO) (2011). Global Atlas on Cardiovascular Disease Prevention and Control.

[36] Li P., Xin J., Wang Y., Li G., Pan X., Wang S., Cheng M., Wen T., Wang G., Liu Z. (2015). Association between particulate matter and its chemical constituents of urban air pollution and daily mortality or morbidity in Beijing city. Environ. Sci. Pollut. Res. Int..

[37] Kim D., Sass-Kortsak A., Purdham J.T., Dales R.E., Brook J.R. (2006). Associations between personal exposures and fixed-site ambient measurements of fine particulate matter, nitrogen dioxide, and carbon monoxide in Toronto, Canada. J. Expo. Sci. Environ. Epidemiol..

[38] National Bureau of Statistics of China Basic Statistics on National Population Census in 2013, 2014 and 2015. http://www.stats.gov.cn/tjsj/ndsj/2011/html/d0305e.htm.

[39] Atkinson R.W., Carey I.M., Kent A.J., van Staa T.P., Anderson H.R., Cook D.G. (2013). Long-term exposure to outdoor air pollution and incidence of cardiovascular diseases. Epidemiology.

