# Supplementary material for: Ambient Air Pollution and Out-of-Hospital Cardiac Arrest in Beijing, China
Source: Int J Environ Res Public Health. 2017 Apr 14;14(4):423. doi: 10.3390/ijerph14040423 (PMC5409624; doi:10.3390/ijerph14040423)
Supplement: Supplementary file 1 [file ijerph-14-00423-s001.pdf]

Ambient Air Pollution and Out-of-Hospital Cardiac Arrest in Beijing, China

Ruixue Xia, Guopeng Zhou, Tong Zhu, Xueying Li and Guangfa Wang

Supplementary Materials

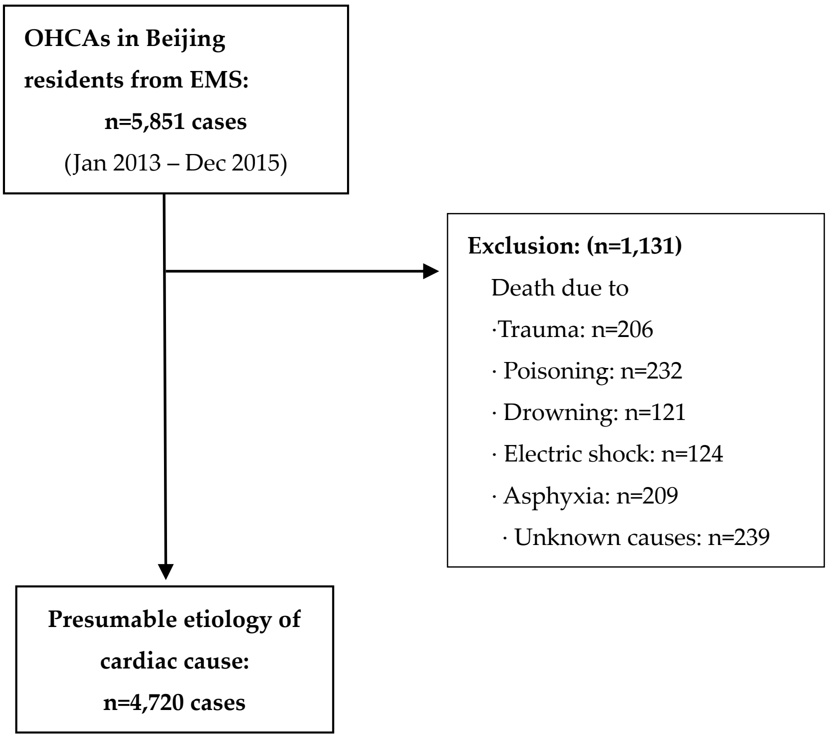

Figure S1: Flow diagram of study population. EMS: emergency medical service; OHCA: out-of-hospital cardiac arrest.

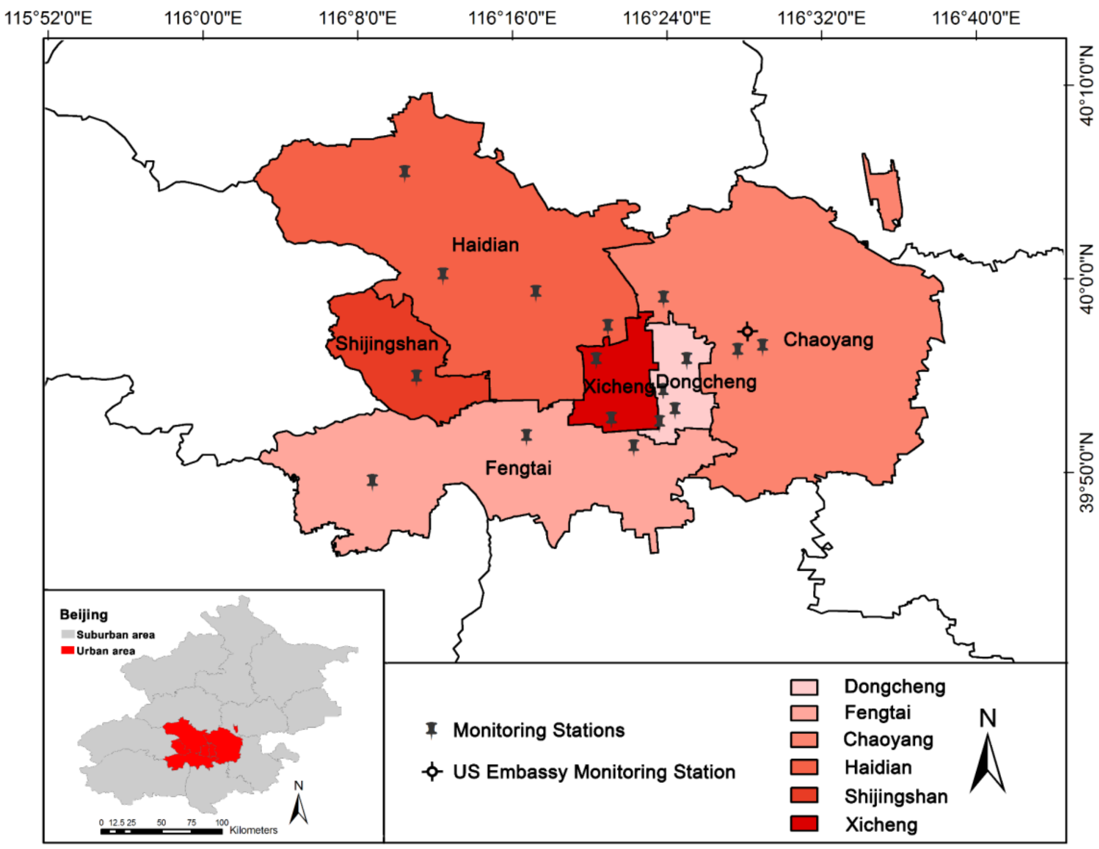

Figure S2. The distribution of all 17 air pollutant monitoring stations and the US Embassy monitoring station located in urban areas in Beijing, China. ArcGIS (ArcMap, version 10.0, ESRI Inc. Redlands, CA, USA) was used to create the map.

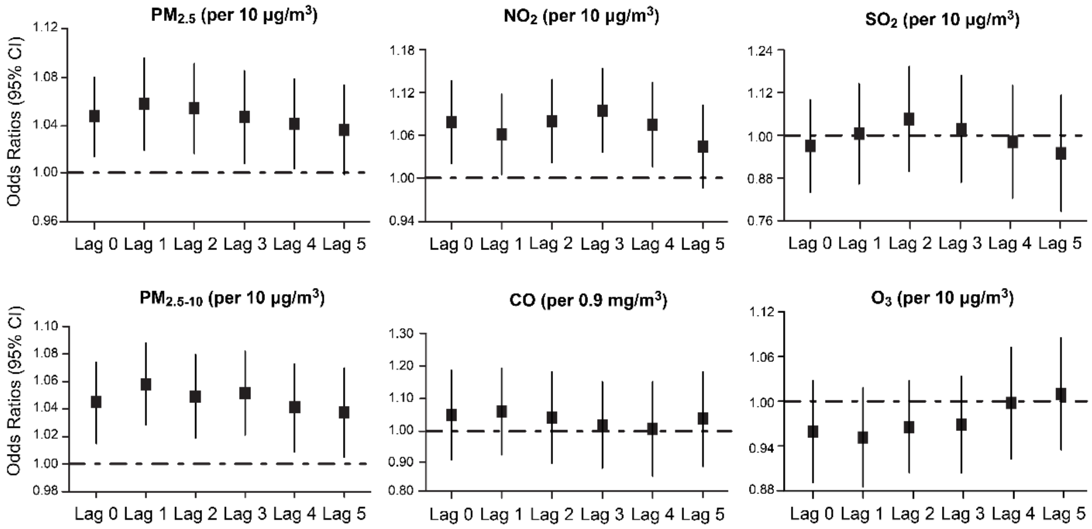

Figure S3. Sensitivity analyses performed using case-crossover models, with estimates from control days matched by temperature to within 0.5 °C. The risk of OHCA in single-pollutant models using various lag lengths. $PM_{2.5}$: particles ≤2.5 μm in aerodynamic diameter; $PM_{2.5-10}$: 2.5≤particles ≤10 μm in aerodynamic diameter; SO_2: sulfur dioxide; NO_2: nitrogen dioxide; CO: carbon monoxide; O_3: ozone.

Table S1. Spearman correlation coefficients among air pollutants, temperature and relative humidity (RH) in Beijing, China, during 2013-2015.

**Variable**	**$PM_{2.5}$**	**$PM_{2.5-10}$**	**SO_2**	**NO_2**	**CO**	**O_3**	**Temperature**	**RH**
$PM_{2.5}$	1.00	0.89 [b]	0.63 [b]	0.58 [b]	0.49 [b]	−0.13	−0.22 [a]	0.23
$PM_{2.5-10}$		1.00	0.51 [b]	0.40 [b]	0.42 [b]	−0.26 [a]	−0.23 [a]	0.30 [a]
SO_2			1.00	0.64 [b]	0.37 [a]	−0.16	−0.21	0.17
NO_2				1.00	0.20	−0.22 [a]	−0.29 [a]	0.21
CO					1.00	−0.15	−0.19	0.26 [a]
O_3						1.00	0.35 [a]	−0.22
Temperature							1.00	−0.25
RH								1.00

[a] $p < 0.05$; [b] $p < 0.05$

Table S2. Estimates of OHCA for 10 μg/m³ moving average of $PM_{2.5}$ concentrations increase in single- and multi-pollutant models at Lag Days 0–5.

	**$PM_{2.5}$ unadjusted**		**$PM_{2.5}$ adjusted for $PM_{2.5-10}$**		**$PM_{2.5}$ adjusted for SO_2**		**$PM_{2.5}$ adjusted for SO_2+NO_2**	
	**OR (95% CI)**	***p***	**OR (95% CI)**	***p***	**OR (95% CI)**	***p***	**OR (95% CI)**	***p***
Lag Day 0	1.05 (1.02,1.08)	<0.01	1.02 (1.00,1.04)	0.02	1.04 (1.02,1.07)	<0.01	1.02 (0.99,1.04)	0.07
Lag Day 1	1.06 (1.03,1.09)	<0.01	1.03 (1.01,1.05)	<0.01	1.05 (1.03,1.08)	<0.01	1.03 (1.00,1.05)	0.03
Lag Day 2	1.04 (1.02,1.07)	<0.01	1.01 (1.00,1.03)	<0.01	1.04 (1.01,1.07)	<0.01	1.03 (0.99,1.06)	0.19
Lag Day 3	1.03 (0.99,1.07)	0.09	1.00 (0.98,1.03)	0.31	1.03 (0.99,1.06)	0.12	1.03 (0.99,1.06)	0.22
Lag Day 4	1.01 (0.99,1.04)	0.21	1.00 (0.98,1.02)	0.43	1.02 (0.99,1.05)	0.16	1.00 (0.98,1.03)	0.38
Lag Day 5	1.00 (0.98,1.03)	0.36	0.99 (0.97,1.02)	0.48	0.99 (0.98,1.02)	0.36	0.99 (0.97,1.02)	0.51

Table S3. Estimates of OHCA for 10 μg/m³ moving average of $PM_{2.5–10}$ concentrations increase in single- and multi-pollutant models at Lag Days 0–5.

	**$PM_{2.5-10}$ unadjusted**		**$PM_{2.5-10}$ adjusted for $PM_{2.5}$**		**$PM_{2.5-10}$ adjusted for SO_2**		**$PM_{2.5-10}$ adjusted for SO_2+NO_2**	
	**OR (95% CI)**	***p***	**OR (95% CI)**	***p***	**OR (95% CI)**	***p***	**OR (95% CI)**	***p***
Lag Day 0	1.04 (1.02,1.06)	<0.01	1.03 (1.00,1.06)	<0.01	1.03 (1.01,1.05)	<0.01	1.01 (1.00,1.03)	0.02
Lag Day 1	1.04 (1.01,1.07)	<0.01	1.03 (1.00,1.07)	<0.01	1.05 (1.02,1.07)	<0.01	1.02 (1.01,1.04)	<0.01
Lag Day 2	1.04 (1.02,1.07)	<0.01	1.02 (0.99,1.05)	0.06	1.04 (1.02,1.06)	0.04	1.01 (1.00,1.01)	0.01
Lag Day 3	1.03 (0.99,1.07)	0.22	1.00 (0.98,1.03)	0.19	1.03 (1.01,1.06)	0.02	1.00 (0.99,1.02)	0.12
Lag Day 4	1.03 (0.99,1.06)	0.18	0.99 (0.96,1.03)	0.31	1.02 (0.99,1.04)	0.20	1.00 (0.98,1.02)	0.18
Lag Day 5	1.02 (0.98,1.05)	0.25	0.98 (0.97,1.02)	0.38	1.00 (0.98,1.03)	0.37	0.99 (0.97,1.03)	0.33
